# Editorial: Biomechanics, Health, Disease and Rehabilitation

**DOI:** 10.3390/bioengineering12060573

**Published:** 2025-05-27

**Authors:** Redha Taiar

**Affiliations:** Department of Sport Sciences, Université de Reims Champagne-Ardenne, MATériaux et Ingénierie Mécanique (MATIM), CEDEX 2, 51687 Reims, France; redha.taiar@univ-reims.fr

## 1. Introduction

The purpose of this Special Issue is to specify and understand the impact of bioengineering in carrying data permitting the comprehension of human behavior in daily life tasks. Today, the application of engineering technologies in patients during rehabilitation training is important as it allows us to explore, understand, and optimize biological problems. Our objective is to summarize the most important methods influencing human rehabilitation performance related to the health sciences for all age groups throughout their lives. In this Special Issue, we encourage papers that aim to promote the latest research in the fields of health, quality of life improvement, and sport rehabilitation and to summarize the best recommendations, as well as papers that help to prevent functional decline and frailty following a life course perspective approach through the utilization of the latest research applied to health in general and applications targeted to all stages of life aimed at the prevention, improved performance, and management of diseases. Modeling, simulation, quantification, and computing of the musculoskeletal system allow us to quantify and improve the parameters characterizing movement in different cases, such as patients’ daily lives. The aim is to effectively combine and coordinate research and results in order to understand and improve human movement in medicine.

In this Special Issue, we share ideas, approaches, opinions, and comments regarding the latest research providing evidence on the mechanisms and safety of rehabilitation exercise and strategies to improve an individual’s quality of life.

In this Special Issue, thirteen papers are included which address those questions. Eleven of them are original research and two are literature reviews that deepen our understanding of these interventions and their practical applications. Herein, we summarize their major contributions according to the subject categories. In the first original research paper, Arianna Carnevale et al. [1] investigate the performance of a custom virtual reality application (VR app) with a stereophotogrammetric system considered the gold standard. The results for the first and second rounds of flexion and abduction displayed low total mean absolute error values and low numbers of unmet conditions. In the second original research paper, Stanislav Štuhec et al. [2] provide an additional analysis and commentary on the men’s 100 m world record of 9.58 s, set by Usain Bolt in the 2009 Berlin World Championships in Athletics. Their biomechanical analysis showed that for Bolt, he completed two phases in this world-record 100 m sprint: acceleration and top velocity. They claim that the maximum velocity phase is the most critical determinant of the final race time. In the third original research paper, Xiangli Gao et al. [3] analyze the gait biomechanics of Latin dancers and non-dancers. They enlisted 21 female Latin dancers and 21 subjects based on specific criteria. The results showed that dancers’ enhanced balance was attributed to external ankle rotation for dance stability, coupled with augmented Achilles tendon and patellofemoral joint strength from prolonged practice. In the fourth original research paper, Han Li et al. [4] aimed to intervene in hearing-impaired children through 12 weeks of cha-cha dance training, evaluating its effects on their PF-related indicators, thus providing a scientific experimental basis for hearing-impaired children to participate in dance exercises effectively. The results of this study showed that after 12 weeks of cha-cha dance training, the PF levels of hearing-impaired students in lower-body strength, flexibility, core strength, and cardiorespiratory endurance were effectively improved. In the fifth original research paper, Axel Klemeit et al. [5] present a biomechanical study aimed at demonstrating the effect of a pin angulation in the monolateral fixator using a composite cylinder model. The authors considered this first attempt to show the biomechanical effect of sagittally oriented pin angulation in a monolateral fixator. In the sixth original research paper, Céline Knopfli et al. [6] hypothesize that there is a correlation between subject-specific musculoskeletal and squat-specific parameters. The results showed a high correlation between the summed volume of the hamstrings and quadriceps and squat depth normalized to thigh length, and a high correlation between leg size and one-repetition maximum load. In the seventh original research paper, Hui-Hsuan Lau et al. [7] investigate the therapeutic advantage of TVM (trans-vaginal mesh) on bladder function by focusing on the thermodynamic workload of voiding. The authors showed that TVM postoperatively lessened the workload of bladder voiding by diminishing voiding resistance, which reduced the pressure gradient required for driving urine flow. In the eighth original research paper, Elizaveta Reganova et al. [8] describe the effects of interval hypoxic training and electrical muscle stimulation (EMS) technology on human productivity. It was found in this paper that EMS training is more likely to cause stress on the body than positively affect cognitive functions. At the same time, interval hypoxic training can be considered a promising direction for increasing human productivity. In the ninth original research paper, Helena Silva-Migueis et al. [9] aimed to understand how upper-limb kinetics behaves alongside activity time during a position-sustained isometric task and how upper-limb kinetics relates to performance fatigability. The results showed that the upper-limb acceleration measured through a single IMU (inertial motion unit) can be a useful and easy strategy for identifying fatigue early. In the tenth original research paper, Tong-Hsien Chow et al. [10] explored the characteristics of static and dynamic plantar pressure profiles with rearfoot posture in elite and recreational badminton players, as well as assessing the transitional changes in plantar loads between static and dynamic states. For elite badminton players, the results showed a possible connection among the static supinated foot, centers of gravity tending towards the right foot, and increased forefoot plantar loads in the dynamic state. In the eleventh original research paper, Jousielle Márcia dos Santos et al. [11] aimed to investigate the impact of WBVT (whole-body vibration training) on oxidative stress markers, plasma irisin levels, and body composition in women with fibromyalgia (FM). The results showed that six weeks of WBVT improves blood redox status markers, increases irisin levels, and reduces visceral adipose tissue mass, favoring less cell damage and improved oxidative balance in women with FM. The study respected the methodology developed in the reporting guidelines for whole-body vibration studies in humans published by Marieke J. G. van Heuvelen et al. and Anika Wuestefeld et al. [12,13]. In the first review paper, Songlin Xiao et al. [14] aimed to summarize research on the effects of tDCS (transcranial direct current stimulation) on foot biomechanics and its clinical applications, and further analyze the underlying ergogenic mechanisms of tDCS. The findings demonstrated that tDCS can improve foot biomechanical characteristics in healthy adults, including proprioception, muscle strength, reaction time, and joint range of motion. In the second review paper, Serena Cerfoglio et al. [15] present a narrative review that aims to present an overview of the state of the art regarding the application of remote motor rehabilitation programs for paucisymptomatic acute and post-acute COVID-19 patients, with a focus on the motor aspects of tele-rehabilitation. The results suggest the feasibility and effectiveness of tele-rehabilitation as a promising tool to complement face-to-face rehabilitation interventions.

## 2. Conclusions

The presented Special Issue “Biomechanics, Health, Disease and Rehabilitation” collectively signifies the evolving landscape of biomechanics and rehabilitation in terms of management of performance and quality of life. We share ideas, approaches, opinions, and comments regarding the latest research providing evidence on the mechanisms and safety of rehabilitation exercise and strategies to improve an individual’s quality of life and sport performance.

## References

[1] Carnevale A., Mannocchi I., Schena E., Carli M., Sassi M.S.H., Marino M., Longo U.G. (2023). Performance Evaluation of an Immersive Virtual Reality Application for Rehabilitation after Arthroscopic Rotator Cuff Repair. Bioengineering.

[2] Štuhec S., Planjšek P., Čoh M., Mackala K. (2023). Multicomponent Velocity Measurement for Linear Sprinting: Usain Bolt’s 100 m World-Record Analysis. Bioengineering.

[3] Gao X., Xu D., Li F., Baker J.S., Li J., Gu Y. (2023). Biomechanical Analysis of Latin Dancers’ Lower Limb during Normal Walking. Bioengineering.

[4] Li H., Kim Y., Zhou Z., Qiu X., Kim S. (2023). Effects of Cha-Cha Dance Training on Physical-Fitness-Related Indicators of Hearing-Impaired Students: A Randomized Controlled Trial. Bioengineering.

[5] Klemeit A., Weber A., Bourauel C., Welle K., Burger C., Schildberg F.A., Deborre C. (2023). The Influence of Sagittal Pin Angulation on the Stiffness and Pull-Out Strength of a Monolateral Fixator Construct. Bioengineering.

[6] Knopfli C., Achermann B., Oberhofer K., Lorenzetti S.R. (2023). First Insights in the Relationship between Lower Limb Anatomy and Back Squat Performance in Resistance-Trained Males and Females. Bioengineering.

[7] Lau H.-H., Lai C.-Y., Hsieh M.-C., Peng H.-Y., Chou D., Su T.-H., Lee J.-J., Lin T.-B. (2023). Pressure–Volume Loop Analysis of Voiding Workload: An Application in Trans-Vaginal Mesh-Repaired Pelvic Organ Prolapse Patients. Bioengineering.

[8] Reganova E., Solovyeva K., Buyanov D., Gerasimenko A.Y., Repin D. (2023). Effects of Intermittent Hypoxia and Electrical Muscle Stimulation on Cognitive and Physiological Metrics. Bioengineering.

[9] Silva-Migueis H., Martínez-Jiménez E.M., Casado-Hernández I., Dias A., Monteiro A.J., Martins R.B., Bernardes J.M., López-López D., Gómez-Salgado J. (2023). Upper-Limb Kinematic Behavior and Performance Fatigability of Elderly Participants Performing an Isometric Task: A Quasi-Experimental Study. Bioengineering.

[10] Chow T.-H., Hsu C.-C., Chen C.-C., Hsu C.-H. (2023). Bipedal Static Supination and Dynamic Forefoot Loading Characteristics in Taiwanese College Badminton Players: A Cross-Sectional Study. Bioengineering.

[11] dos Santos J.M., Taiar R., Ribeiro V.G.C., da Silva Lage V.K., Scheidt Figueiredo P.H., Costa H.S., Pereira Lima V., Sañudo B., Bernardo-Filho M., Sá-Caputo D.d.C.d. (2023). Whole-Body Vibration Training on Oxidative Stress Markers, Irisin Levels, and Body Composition in Women with Fibromyalgia: A Randomized Controlled Trial. Bioengineering.

[12] van Heuvelen M.J.G., Rittweger J., Judex S., Sañudo B., Seixas A., Fuermaier A.B.M., Tucha O., Nyakas C., Marín P.J., Taiar R. (2021). Reporting Guidelines for Whole-Body Vibration Studies in Humans, Animals and Cell Cultures: A Consensus Statement from an International Group of Experts. Biology.

[13] Wuestefeld A., Fuermaier A.B.M., Bernardo-Filho M., da Cunha de Sá-Caputo D., Rittweger J., Schoenau E., Stark C., Marin P.J., Seixas A., Judex S. (2020). Towards reporting guidelines of research using whole-body vibration as training or treatment regimen in human subjects—A Delphi consensus study. PLoS ONE.

[14] Xiao S., Shen B., Zhang C., Xu Z., Li J., Fu W., Jin J. (2023). Effects of tDCS on Foot Biomechanics: A Narrative Review and Clinical Applications. Bioengineering.

[15] Cerfoglio S., Capodaglio P., Rossi P., Verme F., Boldini G., Cvetkova V., Ruggeri G., Galli M., Cimolin V. (2023). Tele-Rehabilitation Interventions for Motor Symptoms in COVID-19 Patients: A Narrative Review. Bioengineering.

